# Lack of association between the Serotonin Transporter Promoter Polymorphism (5-HTTLPR) and Panic Disorder: a systematic review and meta-analysis

**DOI:** 10.1186/1744-9081-3-41

**Published:** 2007-08-18

**Authors:** Carolina Blaya, Giovanni A Salum, Maurício S Lima, Sandra Leistner-Segal, Gisele G Manfro

**Affiliations:** 1 Post-Graduate Program in Medical Sciences, Psychiatry, Universidade Federal do Rio Grande do Sul and Anxiety Disorders Program, Hospital de Clínicas de Porto Alegre, Porto Alegre, Brazil; 2 Associate Professor of Psychiatry, Universidade Católica de Pelotas & Medical Director, Eli Lilly do, Brazil

## Abstract

**Background:**

The aim of this study is to assess the association between the Serotonin Transporter Promoter Polymorphism (5-HTTLPR) and Panic Disorder (PD).

**Methods:**

This is a systematic review and meta-analysis of case-control studies with unrelated individuals of any ethnic origin examining the role of the 5-HTTLPR in PD according to standard diagnostic criteria (DSM or ICD). Articles published in any language between January 1996 and April 2007 were eligible. The electronic databases searched included PubMed, PsychInfo, Lilacs and ISI. Two separate analyses were performed: an analysis by alleles and a stratified analysis separating studies by the quality of control groups. Asymptotic DerSimonian and Laird's Q test were used to assess heterogeneity. Results of individual studies were combined using the fixed effect model with respective 95% confidence intervals.

**Results:**

Nineteen potential articles were identified, and 10 studies were included in this meta-analysis. No statistically significant association between 5-HTTLPR and PD was found, OR = 0.91 (CI95% 0.80 to 1.03, p = 0.14). Three sub-analyses divided by ethnicity, control group quality and Agoraphobia comorbidity also failed to find any significant association. No evidence of heterogeneity was found between studies in the analyses.

**Conclusion:**

Results from this systematic review do not provide evidence to support an association between 5-HTTLPR and PD. However, more studies are needed in different ethnic populations in order to evaluate a possible minor effect.

## Background

Studies in families have shown that Panic Disorder (PD) has a familiar pattern: its prevalence is higher in first degree family members than in control groups [1]. Studies involving siblings show that PD concordance is higher in monozygotic than dizygotic twins [2]. These findings propose that genetic factors contribute to the pathogenesis of PD with an estimated heritability of 30–40% [3], whereas a recent meta-analysis suggests a higher heritability of 48% [4].

Studies on candidate genes for association have been selected on the basis of the molecular therapeutic drugs and panic-provoking agents [5]. For instance, the response shown by panic patients when treated with Serotonin Selective Reuptake Inhibitors (SSRIs) and the worsening when using a serotoninergic agonist suggest a possible serotoninergic dysfunction in this disorder [2,6].

The serotoninergic transporter gene (5-HTT) is located in chromosome 17q11.1-q12 [7] and it codes for a membrane protein that reuptakes serotonin from the synaptic cleft. A size repetition polymorphism has been related to functionality of the serotoninergic transporter protein. The polymorphism is a 44 bp insertion or deletion on the promoter gene region (5 – HTTLPR) resulting in two alleles (l-*long *and s-*short*). The l allele transcription is two or three times more efficient than the s allele [8,9]. The s allele is less active, therefore, resulting in lower serotonin reuptake and, consequently, in increased serotonin in the synaptic cleft [10].

Previous studies found a significant association between s allele and anxiety traits in healthy volunteers [11]. Regarding PD, studies have systematically failed to find any association between this disorder and 5-HTTLPR. This lack of association could be related to the small sample size of studies. However, a recent study has raised the possibility that the l allele could be involved in panic disorder [12].

Although the controversies between 5-HTTLPR in PD may be related to methodological differences between studies, such as ethnicity, another limitation is lack of statistical power [13]. According to Hirschhorn et al. [14], out of 166 studies on gene-disease associations, only six replicated previous findings. Possible causes for this inconsistency include studies with small sample sizes [15,16], as the most realistic genetic association between a polymorphic locus and a disease has been claimed to yield an odds ratio between 1.1 and 1.5 [17]. Thus at least 1000 subjects should be required to detect this association, depending on the prevalence of polymorphism. However, studies typically report sample sizes from 100 to 300 and rarely above 1000 subjects [16,18], justifying the use of meta-analysis to increase power.

The aim of this study is to attempt to answer whether there is an association between the 5-HTTLPR and PD. As the s allele of 5-HTTLPR is significantly involved in anxiety traits, the assumption is that this polymorphism should be involved in PD.

## Methods

### The search

- Electronic databases: studies were identified through PubMed (which encompasses Medline, Premedline, and HelthSTAR), PsychINFO, Lilacs and ISI. The PubMed search was run using the Mesh terms: ("Serotonin Plasma Membrane Transport Proteins" [MeSH] OR "5-HTTLPR" OR "5-HTT" OR "SLC6A4") AND "Panic Disorder" [MeSH]. In PsychINFO, Lilacs and ISI the following words were used: "Panic" AND ("serotonin transporter" OR "serotonergic transporter" OR "5-HTT" OR "5-HTTLPR" OR "SLC6A4"). This search strategy was run in June and rerun in April 2007, and included only human studies, with no language restrictions, and a time scope from January 1996 to April 2007, i.e. since the 5-HTTLPR was described by Heils [9] in 1996.

- Reference cross-checking: the list of references of included studies was searched looking for additional studies.

Contact with authors: efforts were made to contact all research groups of studies included in the analysis to identify unpublished data. Three authors replied and no additional study was identified.

### Inclusion/Exclusion criteria

Studies reporting the 5-HTTLPR in PD patients of any ethnic origin were evaluated by the authors. The inclusion criteria for this systematic review were: (1) type of studies: case-control and family-based studies; (2) type of participants: diagnoses of PD according to standard diagnostic criteria (DSM or ICD); (3) Hardy-Weinberg Equilibrium (HWE). Testing for HWE is commonly used for quality control of large-scale genotyping and is one of the few ways to identify systematic genotyping errors in unrelated individuals [19], and its assumption is required for the allele analysis [20].

Exclusion criteria included (1) studies in which the main disorders were other than PD; (2) replicated data (a part of sample used for more than one publication); (3) insufficient data to perform statistical analysis (unable even after contacting authors). Regarding replicated data, the included study was selected based on the sample size and the availability of information studied in this meta-analysis. Family studies were not included in the analyses because there is only one study published [21], so no analysis could be performed.

**Figure 1:**
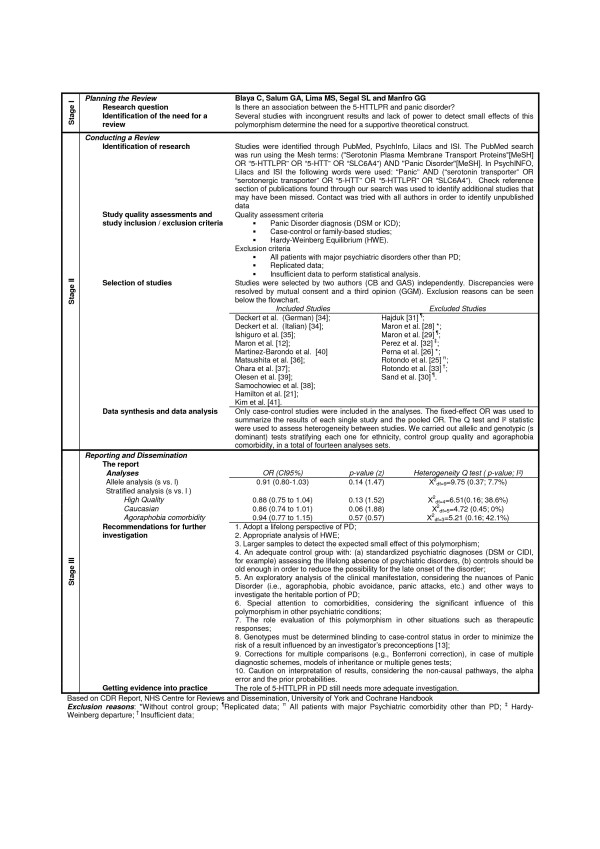
Flowchart – Stages of Systematic Review with Meta-analysis.

This meta-analysis methodology was performed according to MOOSE (*Meta-analysis of Observational Studies in Epidemiology*) group guidelines [22] and stages of this methodology are presented in a flowchart here in (Figure 1).

### Data extraction

Data was independently extracted by two investigators (CB and GAS) using a standardized data extraction form. Discrepancies were resolved by discussion and if consensus was not achieved the decision was made by another reviewer (GGM). Data extracted included number of PD patients and controls for each of the three genotype groups (ll, ls, ss) in each eligible study. Only data regarding 5-HTTLPR and panic disorder were extracted regardless of other polymorphisms, disorders or outcomes reported in the studies. The male/female ratio, mean age, predominant ethnicity of the sample and psychiatric comorbidity were also extracted. Genotype frequencies were used to calculate whether they deviate significantly from HWE. In cases where data was not available in the published reports, authors were contacted directly. The information was classified as not reported when authors did not return to at least three contact trials or did not have this data available.

### Statistical analysis

First, an analysis by alleles was performed because this analysis increases the power to detect differences and is the most used analysis in the current literature. Secondly, we analyze the most used model of inheritance (s dominant) to express directly the ll genotype risk [16]. In order to investigate interaction or confusion between studies, a stratified analysis was run after separating studies by ethnicity, quality of control groups and comorbidity with Agoraphobia, as recommended by Munafo and Flint [15]. It was considered as high quality control groups those with appropriate assessment diagnoses. As we performed fourteen different analyses sets of the same genetic data we also present correct p-values according to Finner's modification of the Bonferroni's procedure for multiple comparisons. Despite being an important analysis, we could not perform a stratified analysis for comorbidity with mood disorders and another anxiety disorders because this data was poorly described in studies included in this meta-analysis. We choose to perform an exploratory analysis because the heritable portion of PD that could be related to this polymorphism is not yet defined.

Asymptotic DerSimonian and Laird's Q test was used to assess heterogeneity. Because tests of heterogeneity may be underpowered to detect heterogeneity between studies when the number of studies is small [23], we also explore heterogeneity quantitatively using the I^2 ^statistic [24].

The results of individual studies, as well as the pooled odds ratio (OR), were synthesized by the fixed effect model with 95% confidence intervals. The significance of the pooled OR was determined by the z test. Publication bias was assessed with a funnel plot (allele analyses). The analyses were done with Review Manager 4.2.8, software developed by the Cochrane Collaboration. The Power Calculator for Genetic Studies software was used in order to estimate the minimum OR that could be detected in this meta-analysis.

## Results

In total, 19 potential publications were identified, and 10 studies were included in this meta-analysis. No analysis was performed with family-based studies because only one was found in our search [21]. One study was excluded because all patients had Bipolar Disorder [25], three studies were excluded because there were no control groups [26-28], three studies were excluded because data were replicated [29-31], one study was excluded due to significant deviation of Hardy-Weinberg equilibrium [32] and one study [33] was excluded due to insufficient data. As one study [34] had two different sub samples clinically assessed with different methodologies, it was considered as two separate studies (Deckert German and Deckert Italian), resulting in ten studies included in our analysis (figure 1). The funnel plot suggests a couple of missing studies with OR higher than 1, but the small number of included trials does not allow drawing conclusions about publication bias.

**Figure 2:**
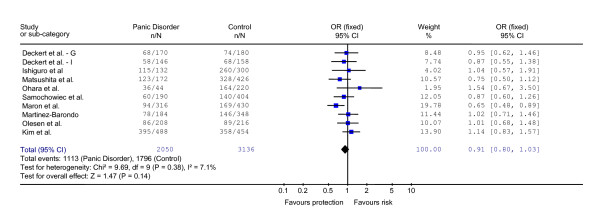
Forest plot with odds ratio of each study and pooled odds ratio for the association between the s allele and risk of Panic Disorder.

Across all 10 case-control studies investigating 5-HTTLPR and panic disorder [12,34-41], a total of 1,025 patients and 1,568 controls were included, totaling 2,050 alleles among patients and 3,136 alleles between controls. Table 1 depicts data extracted from the included studies. Regarding the allele and stratified analyses, no evidence of heterogeneity was found. The statistics of heterogeneity Q test and I^2 ^are shown in the figure 2. We found very similar results between the OR calculated by the random-effects model and Peto OR.

**Table 1:** Included Case-control Studies of 5-HTTLPR and Panic Disorder

							**Genotype Frequency/Total (%)**			**Alelle frequency/Total (%)**		**HWE reported (df = 1)**		
							
**Studies**	**Groups (n)**	**Male (%)**	**Diagnosis**	**Comorbidity**	**Mean Age**	**Origin**	**ss**	**sl**	**ll**	**s**	**l**		**X**^2^	**p**
Ishiguro et al. [35]	*Panic *(66)	30 (45.5)	DSM-IV	NR	40	Japanese	51/66 (77.3)	13/66 (19.7)	2/66 (3)	115/132 (87.1)	17/132 (12.9)	YES	0.986	0.321
	*Control *(150)	70 (46.7)	Not reported		41		114/150 (76)	32/150 (21.3)	4/150 (2.7)	260/300 (86.7)	40/300 (13.3)	YES	0.887	0.346
Matsushita et al. [36]	*Panic (86)*	54 (62.7)	DSM-III-R	NR	37.0	Japanese	44/86 (51.2)	35/86 (40.7)	7/86 (8.1)	123/172 (71.5)	49/172 (28.5)	YES	<0.001	0.992
	*Control (213)*	96 (45.1)	Not reported		37.7		125/213 (58.7)	78/213 (36.6)	10/213 (4.7)	328/426 (77)	98/426 (23)	YES	0.242	0.623
Deckert et al. German [34]	*Panic (85)*	NR	DSM-III-R and CIDI	Yes (Mood) ; Anxiety NR	NR	German	12/66 (14.1)	44/66 (51.8)	29/66 (34.1)	68/170 (40)	102/170 (60)	NO	0.523	0.470
	*Control (90)*	NR	None		NR		16/90 (17.8)	42/90 (46.7)	32/90 (35.6)	74/180 (41.1)	106/180 (58.9)	YES	0.118	0.731
Deckert et al. Italian [34]	*Panic (73)*	NR	DSM-III-R and SADS-LA	Mood absent; Anxiety NR	NR	Italian	13/73 (17.8)	32/73 (43.8)	28/73 (38.4)	58/146 (39.7)	88/146 (60.3)	NO	0.523	0.470
	*Control (79)*	NR	DSM-III-R and DIS^†^		NR		12/79 (15.2)	44/79 (55.7)	23/79 (29.1)	68/158 (43)	90/158 (57)	YES	1.460	0.227
Ohara et al. [37]	*Panic *(22)	11 (50)	DSM-IV	Yes (Anxiety)	35.1	Japanese	14/22 (63.6)	8/22 (36.4)	0/22 (0)	36/44 (81.8)	8/44 (18.2)	NO	1.086	0.769
	*Control *(110)	NR	Not reported		NR		62/110 (56.4)	40/110 (36.4)	8/110 (7.3)	164/220 (74.5)	56/220 (25.5)	NO	0.192	0.661
Samochowiec et al. [38]	*Panic (95)*	24 (23.8)	CIDI	Yes (Anxiety)	38.7	Polonaise	10/95 (10.5)	40/95 (42.1)	45/95 (47.4)	60/190 (31.6)	130/190 (68.4)	NO	0.062	0.803
	*Control (202)*	54 (26.7)	ICD-10		35.9		22/202 (10.9)	96/202 (47.5)	84/202 (41.6)	140/404 (34.7)	264/404 (65.3)	NO	0.492	0.483
Maron et al. [12]	*Panic *(158)	32 (20.2)	DSM-IV MINI	Yes (Anxiety and Mood)	38.0	Estonian	11/158 (7)	72/158 (45.6)	75/158 (47.5)	94/316 (29.7)	222/316 (70.3)	YES	1.288	0.256
	*Control *(215)	56 (26)	MINI^†^		39.8		34/215 (15.8)	101/215 (47)	80/215 (37.2)	169/430 (39.3)	261/430 (60.7)	YES	0.051	0.822
Olesen et al. [39]	*Panic *(104)	28 (26.9)	DSM-IV	Yes (Anxiety)	NR	Danish	15/104 (14.4)	56/104 (53.8)	33/104 (31.7)	86/208 (41.3)	122/208 (58.7)	NO	1.263	0.261
	*Control *(108)	30 (27.7)	Clinical interview		NR		18/108 (16.7)	53/108 (49.1)	37/108 (34.3)	89/216 (41.2)	127/216 (58.8)	NO	0.018	0.894
Martinez-Barondo et al. [40]	*Panic *(92)	28 (30.4)	DSM-IV	NR	35.8	Spanish	18/92 (19.6)	42/92 (45.7)	32/92 (34.8)	78/184 (42.4)	106/184 (57.6)	NO	0.392	0.531
	*Control *(174)	67 (38.5)	Not reported		38.4		36/174 (20.7)	74/174 (42.5)	64/174 (36.8)	146/348 (42)	202/348 (58)	NO	2.798	0.094
Kim et al. [41]	*Panic *(244)	143 (58.6)	DSM-IV	Yes (Mood)	36.1	Korean	159/244 (65.2)	77/244 (31.6)	8/244 (3.3)	395/488 (80.9)	93/488 (19.1)	YES	0.1278	0.721
	*Control *(227)	102 (44.9)	Clinical Interview		33.1		141/227 (62.1)	76/227 (33.5)	10/227 (4.4)	358/454 (78.9)	96/454 (21.1)	YES	0.0035	0.953

Note: NR, Not reported; HWE, Hardy Weinberg Equilibrium; DSM-IV, Diagnostic and Statistical Manual of Mental Disorders IV; ICD-10, International Classification of Diseases; CIDI, Composite International Diagnostic Interview; MINI, MINI International Neuropsychiatric Interview.

^†^Controls with first degree relatives without psychiatric disorders.

In the first analysis, including 5,186 alleles, we compared the 5-HTTLPR allelic distribution between patients with PD and controls of all ten studies included. The pooled OR = 0.91 was not significant (CI95% 0.80 to 1.03; z = 1.47; p = 0.14). The results of this analysis are shown in the forest plot (figure 2). Regarding family studies, we only identified one in this search [21]. The study had evaluated 340 individuals in 45 families with at least three affected people. No linkage between the 5-HTTLPR and PD was observed (p = 0.40).

As the included studies have subjects with different ethnicity and control groups with different quality, we performed stratified analyses. Only five studies – Maron et al. [12], Deckert Italian [34], Samochowiec et al. [38], Olesen et al. [39] and Kim et al. [41] – were classified as having high quality controls. Results showed no significant association between the s allele and PD in both analyses (high and low quality). Regarding the ethnic stratified analysis, no significant association was found in Caucasian or Asian separated analyses. Some studies evaluated the comorbidity with Agoraphobia in their exploratory analysis [12,35,38,41] and no significant association was found in this stratified analysis. Table 2 depicts all these stratified analyses for the allelic analysis and genotype analysis.

**Table 2:** Results of the overall studies and sub-group studies stratified by ethnicity, quality of control group and comorbidity with Agoraphobia

	n^a^	OR (95% CI)	Significance			Heterogeneity		
			
			Z	p-value	Finner's p	χ^2c^	p-value	I^2^
**Allelic analyses (s/l)**^b^	**10**	**0.91 (0.80–1.03)**	**1.47**	**0.14**	**0.58**	**9.75**	**0.37**	**7.7%**
**Ethnicity**								
Caucasians	6	0.86 (0.74 – 1.01)	1.88	0.06	0.58	4.72	0.45	0%
Asians	4	1.02 (0.81 – 1.27)	0.14	0.89	0.92	3.67	0.30	18.3%
**Quality of Control Group**								
High quality control group	5	0.88 (0.75 – 1.04)	1.52	0.13	0.58	6.51	0.16	38.6%
Low quality control group	5	0.96 (0.78 – 1.17)	0.43	0.67	0.76	2.87	0.58	0%
**Agoraphobia comorbidity**								
With Agoraphobia	4	0.94 (0.77 – 1.15)	0.57	0.57	0.69	5.21	0.16	42.4%
Without Agoraphobia	3	0.80 (0.60 – 1.08)	1.48	0.14	0.58	2.30	0.32	12.9%
**Genotype (ls+ss) vs. ll**	**10**	**0.87 (0.71 – 1.06)**	**1.39**	**0.17**	**0.58**	**7**	**0.64**	**0%**
**Ethnicity**								
Caucasians	6	0.85 (0.68 – 1.05)	1.48	0.14	0.58	4.36	0.50	0%
Asians	4	1.01 (0.55 – 1.86)	0.03	0.98	0.98	2.56	0.46	0%
**Quality of Control Group**								
High quality control group	5	0.80 (0.63 – 1.03)	1.76	0.08	0.58	3.72	0.45	0%
Low quality control group	5	1.02 (0.71 – 1.46)	0.11	0.91	0.93	2.30	0.68	0%
**Agoraphobia comorbidity**								
With Agoraphobia	4	0.80 (0.57 – 1.11)	1.32	0.19	0.58	2.90	0.41	0%
Without Agoraphobia	3	0.85 (0.51 – 1.40)	0.65	0.51	0.67	1.18	0.55	0%

Abbreviations: OR, odds ratio (fixed effect); Q, Cochran's x^2^-based Q statistic test used to assess the heterogeneity; z test used to determine the significance of the overall OR; I^2^, inconsistency; Finner's p-value, adjusted p-value for multiple comparison

^a ^The number of studies included are indicated

^b ^The first allele is the risk allele

^c ^Degrees of freedom = number of studies - 1.

We estimated the minimum OR that could be detected in this meta-analysis by allelic analysis. Considering a power of 90%, a PD prevalence of 1.8% in Caucasoid [42], a mean frequency of s allele of 0.45 in a joint analysis with the Power Calculator for genetic studies, the minimum OR that we are capable to found is 1.14.

## Discussion

This is the first systematic review between the 5-HTTLPR and PD and no statistically significant association was found. In the overall analysis, there was no evidence for heterogeneity among the studies. This indicates no greater variation among the studies than could be expected by chance and provides the validity to the meta-analysis by suggesting that studies included in this analysis are comparable. However, no association was found between this polymorphism and PD even in all stratified analyses.

The main problem in genetic studies and psychiatric disorders is probably the lack of a phenotype definition. Clinical diagnoses according to DSM-IV may be heterogeneous constructs that combine elements with distinct genetic influences [43]. Additionally, psychiatric disorders usually overlap, and comorbidity might be a bias that impairs some associations. Therefore, some authors are interested in defining "intermediate" phenotypes that might have more direct expression of genes influencing a complex disorder and may have a simpler genetic architecture [43].

Also, a recently detected genetic variation (16A allele) [44] in the long allele of 5-HTTLPR has been linked to a lower expression of the serotonin transporter [45,46]. No study has evaluated this polymorphism neither in panic disorder nor in anxiety traits. Hence, studies should combine the evaluation of different polymorphisms that influence the protein expression.

### Limitations of this study

The publication bias could not be adequately assessed in our meta-analysis by the funnel plot due to the small number of studies available. However, we believe that our search strategy was comprehensive enough. Moreover, additional sources of trials like contacting authors of included trials yield no additional study.

The sample size found in our meta-analysis is below that which is required to identify associations with minor effects [17]. In the sub-group analysis the sample size became even smaller. Thus, further investigation using larger sample sizes, in which the control group is properly diagnosed and ethnicity is evaluated, is needed. Such studies should focus on exploratory analyses in order to identify the heritable features that might be related to this polymorphism. Additionally, we could not perform a sub-group analysis divided by gender, because only one study reported this data. Neither did we find any stratified analyses regarding co-morbidity (with alcohol, mood and anxiety disorders), even though associations between the 5-HTTLPR and these psychiatric disorders have been reported [30,47,48].

This meta-analysis was carried out with case-control studies. However, some authors [13] suggest that the results from this design finding a positive association between the genome and a disorder could be false. Additionally, a positive result from a case-control study might be due to population stratification rather than linkage disequilibrium [49] affected by the different disease prevalence and different marker frequencies in the subpopulation thereby producing spurious associations [50,51]. The use of Genomic Control, although with limitations [52], may be an alternative in analyzing data of case-control studies. Unfortunately, we could not perform an additional analysis with family studies because we identified only one study with this design. Family-based studies have a unique design in which population stratification is controlled.

## Conclusion

In summary, results from this systematic review do not support the hypothesis of a significant association between 5-HTTLPR and PD. All the sub-analyses performed failed to find an association between PD and this polymorphism. However, more studies are needed in different ethnic populations in order to evaluate a possible minor effect. Finally, the 5-HTTLPR does not seem to play a major role in the genetics of panic disorder and, therefore, other polymorphisms should be investigated.

## Abbreviations

PD, Panic Disorder

SSRIs, Serotonin Selective Reuptake Inhibitors

5-HTTLPR, Serotonin Transporter Promoter Polymorphism

DSM, Diagnostic and Statistical Manual

ICD, International Classification of Diseases

CIDI, Composite International Diagnostic Interview

MINI, MINI International Neuropsychiatric Interview.

5-HTT, Serotoninergic transporter gene

SLC6A4, Solute Carrier Family 6 (Neurotransmitter transporter serotonin), member 4

HWE, Hardy-Weinberg Equilibrium

MOOSE, Meta-analysis of Observational Studies in Epidemiology

OR, Odds ratio

CI95%, Confident Interval of 95%

## Competing interests

Mauricio S. Lima is medical director of Eli Lilly do Brazil.

Carolina Blaya and Gisele G. Manfro are speakers of Eli Lilly Brazil.

Giovanni A. Salum and Sandra Leistner-Segal have no competing interests.

## Authors' contributions

CB conceived of the study, extracted the data, participated in the design and drafted the manuscript. GAS extracted the data, participated in the design, drafted the manuscript and performed the statistical analysis. MSL helped in the statistical analysis and helped to draft the manuscript. SLS helped to draft the manuscript. GGM, participated in its design, coordination and drafted the manuscript. All authors read and approved the final manuscript.
